# On-surface synthesis of nitrogen-doped nanographene with an [18]annulene pore on Ag(111)

**DOI:** 10.1038/s42004-023-01023-z

**Published:** 2023-10-20

**Authors:** Kewei Sun, Donglin Li, Takahito Kaihara, Satoshi Minakata, Youhei Takeda, Shigeki Kawai

**Affiliations:** 1 International Center for Young Scientists, National Institute for Materials Science, 1-2-1 Sengen, Tsukuba, Ibaraki 305-0047 Japan; 2 Center for Basic Research on Materials, National Institute for Materials Science, 1-2-1 Segen, Tsukuba, Ibaraki 305-0047 Japan; 3 Department of Applied Chemistry, Graduate School of Engineering, Osaka University, Yamadaoka 2-1, Suita, Osaka 565-0871 Japan; 4 Graduate School of Pure and Applied Sciences, University of Tsukuba, Tsukuba, 305-8571 Japan

**Keywords:** Scanning probe microscopy, Structural properties, Synthesis of graphene

## Abstract

On-surface synthesis is of importance to fabricate low dimensional carbon-based nanomaterials with atomic precision. Here, we synthesize nitrogen-doped nanographene with an [18]annulene pore and its dimer through sequential reactions of debromination, aryl–aryl coupling, cyclodehydrogenation and C–N coupling on Ag(111) from 3,12-dibromo-7,8-diaza[5]helicene. The inner structures of the products were characterized with scanning tunneling microscopy with a CO terminated tip at low temperature. Furthermore, the first four unoccupied electronic states of the nanographene were investigated with a combination of scanning tunneling spectroscopy and theoretical calculations. Except for the LUMO + 2 state observed at +1.3 V, the electronic states at 500 mV, 750 mV and 1.9 V were attributed to the superatom molecular orbitals at the [18]annulene pore, which were significantly shifted towards the Fermi level due to the hybridization with the confined surface state.

## Abstract (web summary)

On-surface synthesis is a key tool to access low dimensional carbon-based nanomaterials with atomic precision. Here, the authors synthesize a nitrogen-doped nanographene structure with an [18]annulene pore on Ag(111) through sequential debromination, aryl–aryl coupling, cyclodehydrogenation and C–N coupling reactions from a 3,12-dibromo-7,8-diaza[5]helicene precursor.

## Introduction

Nanographenes (NGs) can be regarded as representative zero-dimensional graphene derivatives and have attracted significant attention from researchers in recent years. The nanomaterials are promising candidates for forthcoming carbon-based nanoelectronics^1^, spintronics^2^, solar cells^3^, and gas storages^4^ because their electronic, optical and magnetic properties can be tuned by edge topologies and sizes^5–8^ as well as heteroatom substitutions^9^. Specifically, engineering band gaps of NGs would lead to various applications such as photovoltaic system^10^ and light-emitting diode (LED)^11^. On-surface synthesis as a bottom-up approach became a powerful method to obtain these nanocarbon materials because of the high-controllability of the structure down to atomic scale^12,13^. The small molecules synthesized in wet chemistry are usually sublimated on metal surfaces, and subsequently annealed to activate the reaction. Through homo-^14–18^ and hetero-coupling^19–22^ and dehydration^23^ of molecules, desired products are obtained. This method is free from the solubility issue, which often limits the type of reactants in wet chemistry^8,24^. Adsorption to the metal substrate also lowers the activation barrier of cyclodehydrogenation for planarization^25–27^, which usually requires harsh conditions in wet chemistry. In the last decade, the on-surface synthesis has been successfully developed to fabricate carbon-based low dimensional nanostructures with atomic precision such as graphene nanoribbons^25,28–36^ and covalent organic frameworks^22,23,37,38^. Besides one- and two-dimensional nanocarbons, NGs with various edge shapes and sizes have also been obtained by dehalogenative coupling or cyclodehydrogenation^26,27,39–42^. For instance, NGs with sub-nanoscale pores^43–47^ and with heteroatom substitutions^48–51^ were successfully synthesized. However, on-surface synthesis of NGs, having both the pore and the heteroatom substitution, is still scarce.

Here, we employ 3,12-dibromo-7,8-diaza[5]helicene (DBDH, **1**) molecule^52^ as a precursor to synthesize nitrogen-doped NG and its dimer with sub-nanoscale pores on Ag(111). Triangular organometallic compounds composed of three molecular units are first synthesized via dehalogenative homo-coupling by annealing at 100 °C. Higher temperature annealing at 150 °C induces the cleavage of C–Ag bonds and the subsequent formation of the C–C bonds between the units. Finally, the nitrogen-doped NG with an [18]annulene pore is obtained via the planarization through cyclodehydrogenation at 250 °C. Further annealing at 300 °C leads to the formation of the dimer by C–N coupling. Scanning tunneling microscopy (STM) with a CO-terminated tip reveals the structures of the products. We also measure the first four lowest unoccupied molecular orbital (LUMO) states with scanning tunneling spectroscopy (STS). Among them, three unoccupied states mainly located around the [18]annulene pore are significantly downshifted towards the Fermi level from the energies of the molecular orbitals in vacuum, which are obtained by density functional theory (DFT) calculations.

## Results and discussions

### Scheme of reaction processes of molecule 1 on Ag(111)

**Fig. 1:**
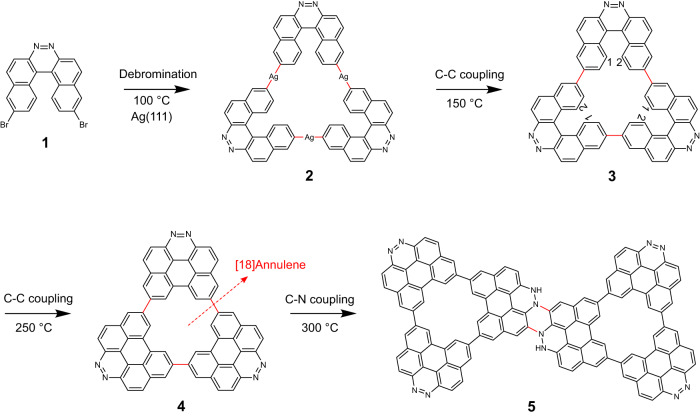
Scheme of reactions of molecule **1**. Reaction processes to form a nitrogen-doped nanographene with an [18]annulene pore and its dimer on Ag(111).

The helicene derivative **1** (Fig. 1) is employed to synthesize the nitrogen-doped NG on Ag(111). The precursor is non-planar due to the steric hindrance. The organometallic triangular structure is first formed with three DBDH molecules by formation of aryl–Ag–aryl bonds through debromination at 100 °C, namely **2**. Then, the C–C bond between the units is formed via aryl–aryl coupling at 150 °C, namely **3**. Finally, **3** is planarized by cyclodehydrogenation (C1–C2 coupling) at 250 °C, resulting in the formation of nitrogen-doped NG with an [18]annulene pore, namely **4**. The nanographenes are further fused to each other by annealing at 300 °C, and consequently the dimer **5** was formed.

### Synthesis of nitrogen-doped NG with an [18]annulene pore

**Fig. 2:**
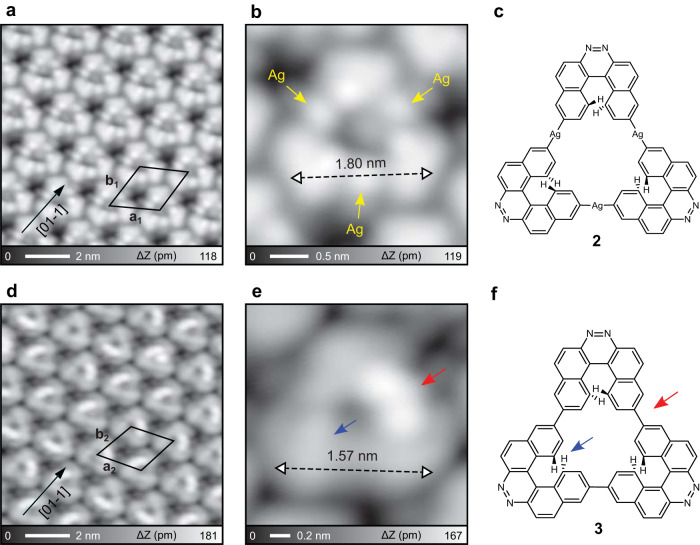
Debromination and C–C coupling of **1**. **a** STM topography of the products after annealing at 100 °C. **b** Close-up view of the topography and (**c**) the corresponding chemical structure. **d** STM topography of the products after annealing at 150 °C. **e** Close-up view of the topography and (**f**) the corresponding chemical structure. Measurement parameters: sample bias voltage *V* = 50 mV and tunneling current *I* = 10 pA in **a**. *V* = 200 mV and *I* = 10 pA in **b**. *V* = 200 mV and *I* = 3 pA in **d**. *V* = 200 mV and *I* = 5 pA in **e**.

Molecules **1** were deposited on a clean Ag(111) surface held at room temperature, and the sample was subsequently annealed at 100 °C. We found that ordered uniform triangular nanostructures extended over the terrace with periodicities of *a*_1_ = 2.29 ± 0.01 nm and *b*_1_ = 2.19 ± 0.01 nm (Fig. 2a). The triangular structure was composed of three units, whose length of one side was 1.80 ± 0.02 nm as indicated by a double arrow. The units were connected via three bright dots as marked by yellow arrows in Fig. 2b, which correspond to single silver atoms^53^. Thus, the annealing temperature of 100 °C was high enough to cleave the aryl-Br bonds in **1** on Ag(111)^54,55^, resulting in the formation of the C–Ag–C organometallic bond (**2**, Fig. 2c). Due to the steric hinderance in the helicene structure, one side of the unit protruded more from the surface than another. Subsequently, the organometallic compound has a chiral configuration on Ag(111). We found that each self-assembled molecular island was composed of single chiral molecules (Fig. S1). To induce the further reaction, the sample was annealed at 150 °C. Although each unit still has a triangular shape, the self-assembly became more close-packed as the periodic parameters of *a*_2_ = 1.97 ± 0.01 nm and *b*_2_ = 1.95 ± 0.01 nm (Fig. 2d). The close-up view of the STM topography shows a significant change in the unit (Fig. 2e). The bright spots (Ag atoms) between the units disappeared, and the length of one side reduced to 1.57 ± 0.06 nm as indicated by a double arrow. Thus, these results indicate that the aryl–Ag–aryl bond was transformed to the aryl–aryl bond^54,55^, namely the Ullmann-type reaction. The slight distorted part as indicated by a blue arrow implies that no cyclodehydrogenation has occurred. The side of the triangle indicated by a red arrow significantly protruded from the surface. We attributed that the non-planar structure was caused by upward shifting of the naphthyl moieties in the helicenes (Fig. S2 and Supplementary Data 1). This structure is named as **3** (Fig. 2f).

**Fig. 3:**
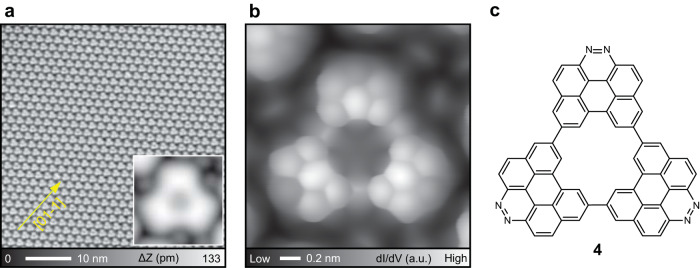
Cyclodehydrogenation towards synthesis of **4**. **a** Large-scale STM topography of the nitrogen-doped NGs on Ag(111). The inset shows a close-view image of single molecule. **b** Constant height d*I*/d*V* mapping of the product and (**c**) the corresponding chemical structure. Measurement parameters: *V* = 200 mV and *I* = 4 pA in **a**. For constant height dI/dV map:*V* = 10 V, *V*_ac_ = 10 mV in **b**.

In order to planarize **3**, cyclodehydrogenation was induced by further annealing at 250 °C. We found that the extended molecular island was still composed of triangular molecules (Fig. 3a). The inset shows the close-up view of the product, which is planar and has a sub-nanoscale pore in the center. To investigate the structure in detail, the tip apex was terminated by a CO molecule^56,57^. The bond-resolved image shows the skeleton structure, in which all units had the ring closing. We also found that the nitrogen-doped heterocyclic ring appeared dim (Fig. 3b), which is in agreement with previous studies^51,58^. Thus, the observed molecule corresponds to the target product **4**, which has the [18]annulene in the center (Fig. 3c). The subtle bright dots around **4** (Fig. 3b and S3) correspond to dissociated Br atoms adsorbed on Ag(111). It is also worth noting that no successful synthesis of **4** was seen on Au(111) and Cu(111) surfaces. We deduce that the formation of the organometallic triangular intermediate **2** plays a major role in the synthesis of NG **4**. Since the number of adatom on Au(111) is much smaller than that on Ag(111), it is difficult to form the organometallic intermediates on Au(111) (Fig. S4). We found that the selective formation of organometallic chains on Cu(111), which may relate to different lattice distance and reactivity (Fig. S5).

### Electronic properties of nitrogen-doped NG

**Fig. 4:**
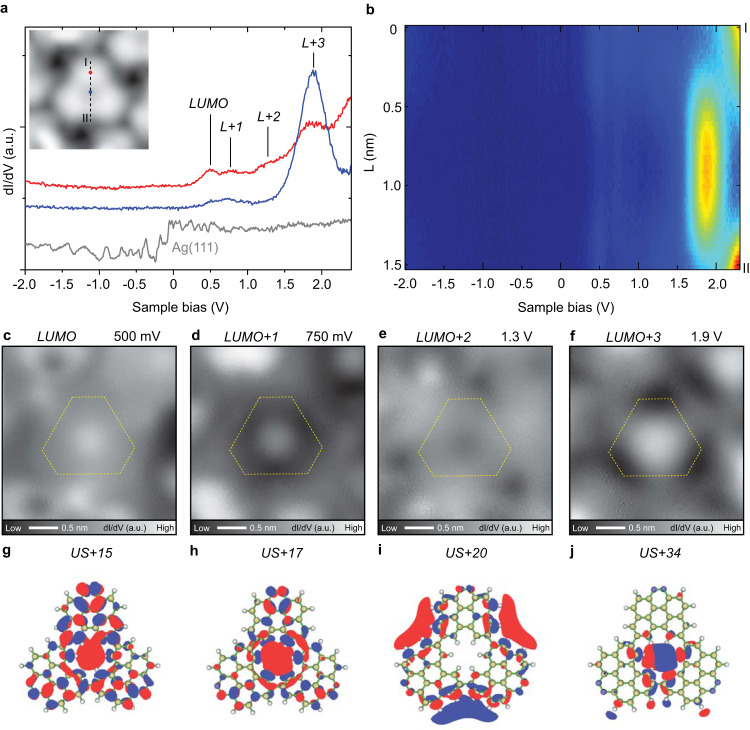
Electronic properties of **4**. **a** d*I*/d*V* curves recorded above the individual **4** (red and blue lines) and the bare Ag(111) substrate (gray). The inset shows a close-up view of **4**. **b** 2D map composed of 51 sequential d*I*/d*V* curves taken along the I-II line indicated in the inset of **a**. Constant current d*I*/d*V* maps taken at different bias voltages: (**c**) 500 mV, (**d**) 750 mV, (**e**) 1.3 V, (**f**) 1.9 V, respectively. **g**–**j** DFT calculated spatial distribution of four unoccupied electronic states on chemical structures of **4**. Measurement parameters: *V* = 10 mV and *I* = 10 pA in the inset of **a**. *V* = 2.4 V, *I* = 300 pA, *V*_ac_ = 10 mV for STS in **a**. *V* = 500 mV, *I* = 110 pA, *V*_ac_ = 10 mV in **c**. *V* = 1.1 V, *I* = 110 pA, *V*_ac_ = 10 mV in **d**. *V* = 1.3 V, *I* = 120 pA, *V*_ac_ = 10 mV in **e**. *V* = 1.9 V, *I* = 150 pA, *V*_ac_ = 10 mV in f.

To investigate the electronic properties, we conducted STS measurements on **4**. CO molecules also adsorb at the [18]annulene pore site (Fig. S6). To exclude the influence of the adsorption on the electronic states of molecules, we conducted STS measurements before dosing CO molecules. The d*I*/d*V* spectra were recorded above the unit, the pore, and the bare Ag(111) surface as a reference, as indicated by red, blue, and gray dots, respectively (Fig. 4a). We found four prominent peaks of empty states (+500 mV, +750 mV, +1.3 V and +1.9 V) in the curves taken above both the unit and pore sites. The spectra have no significant feature below the Fermi energy level, which is most probably due to the suppression of the occupied state by a strong interaction between **4** and the Ag substrate. Here, the first four unoccupied electronic states of **4** are attributed as LUMO, LUMO + 1, LUMO + 2, and LUMO + 3 states. The two-dimensional map composed of 51 sequential d*I*/d*V* curves was taken along the I-II line in the inset of Fig. 4a. A strong signal of the LUMO + 3 state is located at the pore (Fig. 4b). In order to further investigate the spatial distribution of the four electronic states, the d*I*/d*V* maps were recorded at the corresponding bias voltages (Fig. 4c–f). Although the state of 500 mV distributed on the whole molecule, the pore site appeared relatively brighter. Only the pore appeared bright in the d*I*/d*V* map taken at 750 mV while the other parts of the molecule appeared darker. In contrast, the state of 1.3 V distributed only on the molecular backbone and had no significant signal at the pore. Finally, the strong localization of the state was observed in the pore at 1.9 V. In short, the empty states at 500 mV, 750 mV, and 1.9 V were concentrated mostly in the [18]annulene pore. Other nanographene structures with [18]annulene pores also exhibit a high density of empty states in the pores even on Au(111), demonstrating the generality of this phenomenon^43,45,46^. In addition, no significant feature of occupied state was observed (Fig. S7), similar to previous studies^59–61^, which is most probably due to the suppression of the occupied state by a strong interaction between **4** and the Ag substrate^61,62^.

To get an insight into the electronic properties, a number of the unoccupied states of **4** and the corresponding density of states were obtained by DFT calculation in vacuum (Figs. S8 and S9 and Supplementary Data 1). Among them, we found three electronic states that have strong electronic densities in the [18]annulene pores. The unoccupied state (US) + 15 concentrates on both the molecular backbone and the pore (Fig. 4g), which is in agreement with the d*I*/d*V* map measured at 500 mV (Fig. 4c). In contrast, the density of US + 17 state weakened on the molecular backbone while enhanced on the pore (Fig. 4h), generally consistent with the d*I*/d*V* map at 750 mV (Fig. 4d). The US + 34 state exhibits a hexagonal distribution in the pore (Fig. 4j), fairly agrees with the d*I*/d*V* map at 1.9 V (Fig. 4f). The agreement of the contrasts suggests that these three states are related to the superatom molecular orbitals (SAMOs)^43,60,63^, which are caused by the hybridization of π-orbitals of the carbon backbone within the pore. We also found that the US + 20 state distributes on both the carbon skeleton and the concave regions around the molecule (Fig. 4i), which is also in agreement with the d*I*/d*V* map measured at 1.3 V (Fig. 4e). These calculated four unoccupied states in vacuum have relatively high energies, which are significantly lowered by the strong molecule-silver substrate interaction^63^.

### Synthesis of nitrogen-doped NG dimer

**Fig. 5:**
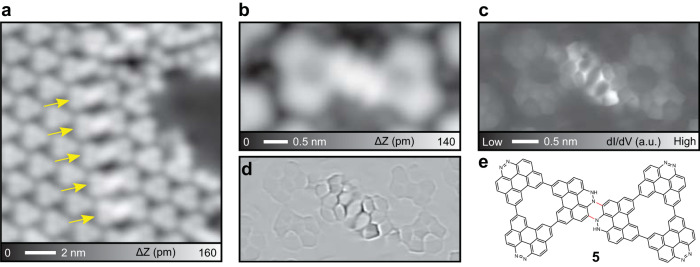
Synthesis of **4** dimers on Ag(111). **a** STM topography of the Ag(111) surface after annealing at 300 °C for 10 min. **b** Close-up view of single dimer. **c** Constant height d*I*/d*V* map over the dimer in **b**, and (**d**) the corresponding Laplace filtered image. **e** Chemical structure of the dimer, **5**. Measurement parameters: *V* = 200 mV and *I* = 10 pA in **a**. *V* = 200 mV and *I* = 5 pA in **b**.

To investigate the stability of **4** on Ag(111), we further heated the sample to 300 °C. Most **4** in the molecular island were still intact (Fig. 5a). Interestingly, we found the dimer-like products as indicated by arrows. A close-up view of the individual product shows that the junction between the units appeared brighter than the other parts (Fig. 5b). The constant height d*I*/d*V* map (Fig. 5c) and the corresponding Laplace filtered image (Fig. 5d) revealed the inner structure, in which two molecules were fused by formation of the pyrazine ring (Fig. 5e). Around the junction, a strong signal in the d*I*/d*V* map was detected. Since no magnetic feature was observed in the d*I*/d*V* curve (Fig. S10), the bright contrast may result from the topographic corrugation caused by the slight sp^3^ character of the central NH–N units. Thus, this dimer is apparently product **5**, which was proposed in Fig. 1. Since N=N double bond often serves as an electrophile in organic chemistry^64^, nucleophilic attach by the π bond of the adjacent monomer compound followed by migration of hydrogen to the adjacent nitrogen can afford NH unit in a reasonable way. Nevertheless, the N core level measurement by XPS measurement could conclude the chemical analysis^65^. Note that no significant difference in the electronic properties between the dimer and the monomer was observed (Fig. S11). After annealing to 350 °C, only disordered nanostructures were formed on Ag(111) (Fig. S12), which is most probably associated with the cleave of C–C bonds of NGs and subsequent random fusion.

## Conclusions

In summary, the nitrogen-doped nanographene with an [18]annulene pore and its dimer were synthesized with 3,12-dibromo-7,8-diaza[5]helicene on Ag(111) through sequential reactions of debromination, aryl–aryl coupling, cyclodehydrogenation, and C–N coupling. The bond-resolved imaging with a CO molecule terminated tip shows the molecular backbones. The STS measurements detected the four unoccupied states of the nitrogen-doped nanographene on Ag(111). Among them, three states concentrated mainly on the [18]annulene pore. These energies significantly lowered from those obtained by DFT calculations in vacuum, which is most probably caused by the strong hybridization with the confined surface state. This work demonstrated that on-surface synthesis is an advanced method to fabricate nanographenes with defined accurate shapes.

## Methods

### STM experiments

All experiments were conducted with a home-made low temperature scanning tunneling microscope (STM), operating under ultrahigh vacuum (<1 × 10^−10^ mbar) at 4.3 K. Clean Ag(111) surfaces were obtained through repeated cycles of Ar^+^ sputtering for 10 min and annealing at 700 K for 10 min. The temperature of sample was measured by a thermocouple and a pyrometer. 3,12-dibromo-7,8-diaza[5]helicene (**1**) molecules^52^ were deposited from Knudsen cells (Kentax GmbH). A STM tip was made from the chemically etched tungsten. For constant height d*I*/d*V* imaging, the tip apex was terminated by a CO molecule^56,57^. The modulation amplitude was 7 mV_rms_ and the frequency was 510 Hz for the STS measurement.

### Theoretical calculations

DFT calculations were performed by the Gaussian 16 package^66^ using the B3LYP functional and 6-31 G(d,p) basis set. The molecular orbitals were further calculated by Multiwfn 3.8 code^67,68^.

### Material synthesis

3,12-Dibromo-7,8-diaza[5]helicence (**1**) was prepared according to the procedure reported in the paper^52^.

### Supplementary information

Supplementary Information

Description of Additional Supplementary File

Supplementary Data 1

## Data Availability

All relevant data are available from the authors upon reasonable request.
