# Supplementary material for: On-surface synthesis of nitrogen-doped nanographene with an [18]annulene pore on Ag(111)
Source: Commun Chem. 2023 Oct 20;6:228. doi: 10.1038/s42004-023-01023-z (PMC10589310; doi:10.1038/s42004-023-01023-z)
Supplement: Supplementary file 1 — Supplementary Information [file 42004_2023_1023_MOESM1_ESM.pdf]

Supplementary Materials for

On-Surface Synthesis of Nitrogen-Doped Nanographene with an [18]Annulene Pore on Ag(111)

Kewei Sun[1], Donglin Li[2], Takahito Kaihara[3], Satoshi Minakata[3], Youhei Takeda[3]*, Shigeki Kawai[2,4]*

[1]*International Center for Young Scientists, National Institute for Materials Science, 1-2-1 Sengen, Tsukuba, Ibaraki 305-0047, Japan.*

[2]*Center for Basic Research on Materials, National Institute for Materials Science, 1-2-1 Segen, Tsukuba, Ibaraki 305-0047, Japan.*

[3]*Department of Applied Chemistry, Graduate School of Engineering, Osaka University, Yamadaoka 2-1, Suita, Osaka 565-0871, Japan.*

[4]*Graduate School of Pure and Applied Sciences, University of Tsukuba, Tsukuba 305-8571, Japan.*

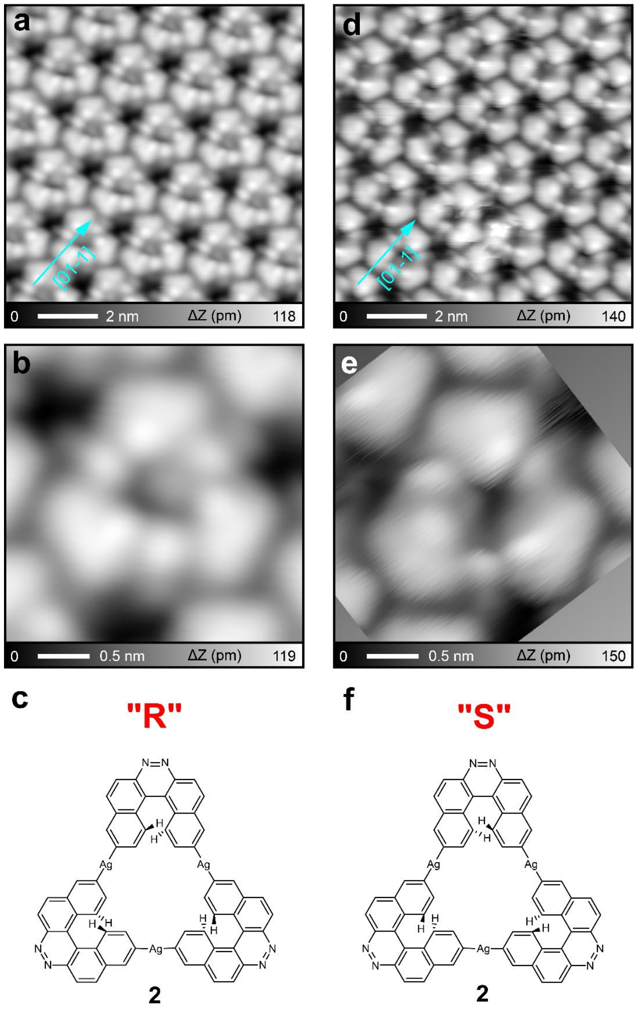

Figure S1. The chirality of **2** on Ag(111). (a) STM topography of assembled **2**. (b) A close-view image of individual **2**. (c) The corresponding chemical structure of **2** with “R” chirality. STM images in (a), (b) are identical with Figure 1a, b. (d) STM topography of assembled **2**. (e) A close-view image of individual **2**. (f) The corresponding chemical structure of **2** with “S” chirality. Measurement parameters: Sample bias voltage $V = 50$ mV and tunneling current $I = 10$ pA in (a). $V = 200$ mV and $I = 10$ pA in (b). $V = 600$ mV and $I = 10$ pA in (d). $V = 500$ mV and $I = 10$ pA in (e).

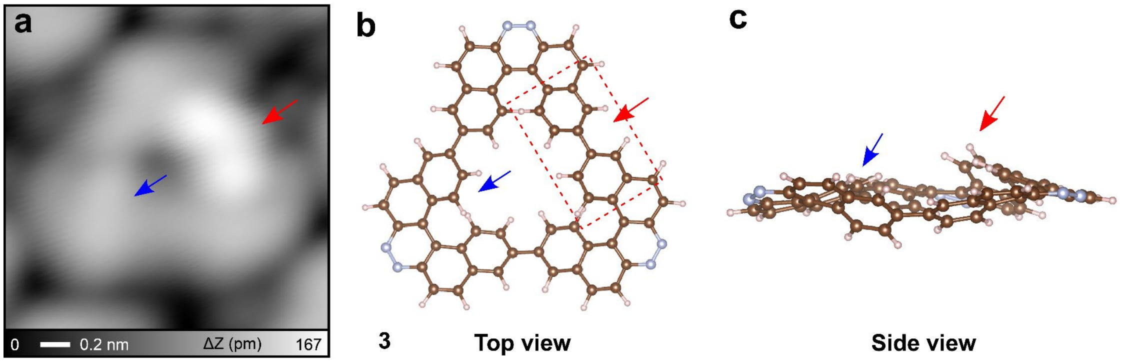

Figure S2. Calculated chemical structure of **3**. (a) STM topography of individual **3**. (a) is identical with Figure 1e. Optimized chemical structure of **3** without consideration of substrate by DFT calculations: (b) top view; (c) side view. The two naphthyl moieties of helicene (indicated by dashed rectangle in (b) and red arrow in (c)) are much out-of-plane, showing bright features (STM topography in (a)). Measurement parameters: $V = 200$ mV and tunneling current $I = 5$ pA in (a).

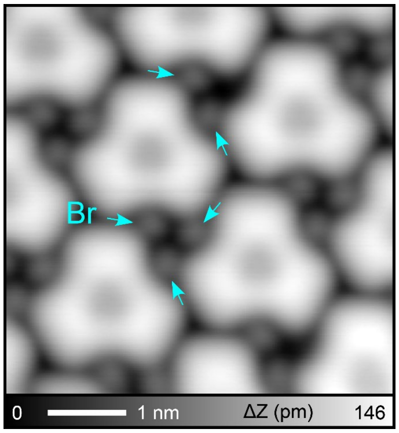

Figure S3. STM topography of nitrogen-doped NGs **4** and Br atoms on Ag(111). Measurement parameters: $V = 200$ mV and $I = 10$ pA.

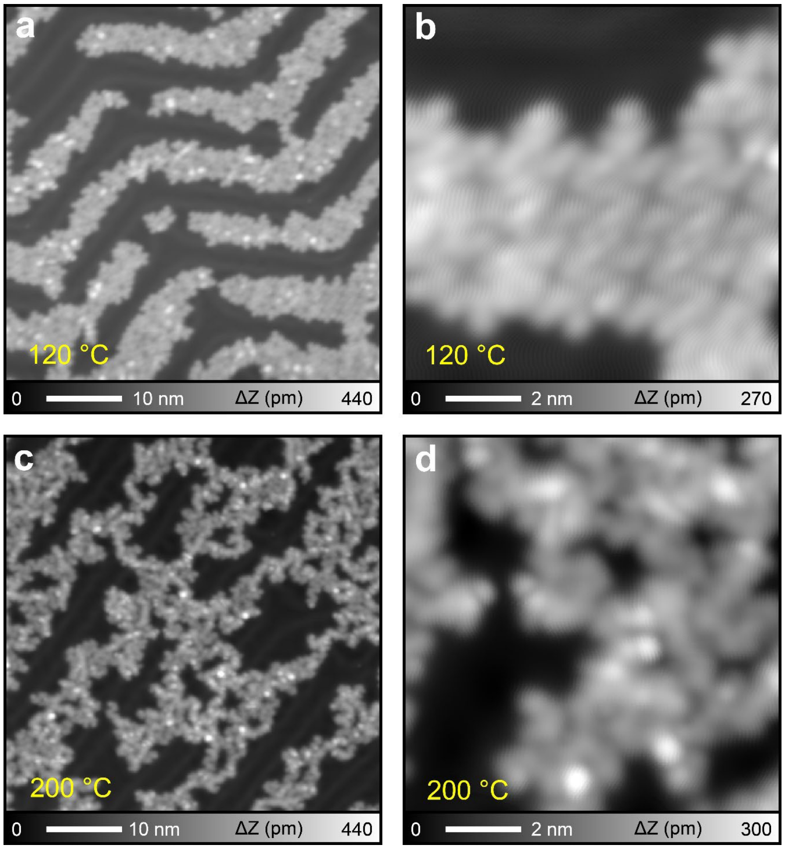

Figure S4. On-surface reactions of **1** on Au(111). (a) Large-scale STM topography of the sample after annealing at 120 °C and (b) its close-up view. (c) Large-scale STM topography of the sample after annealing at 200 °C and (d) its close-up view. Only disordered structures were observed on Au(111) after annealing to 120 °C and 200 °C. Measurement parameters: V = 200 mV and I = 10 pA in (a). V = 100 mV and I = 20 pA in (b). V = 200 mV and I = 20 pA in (c). V = 200 mV and I = 50 pA in (d).

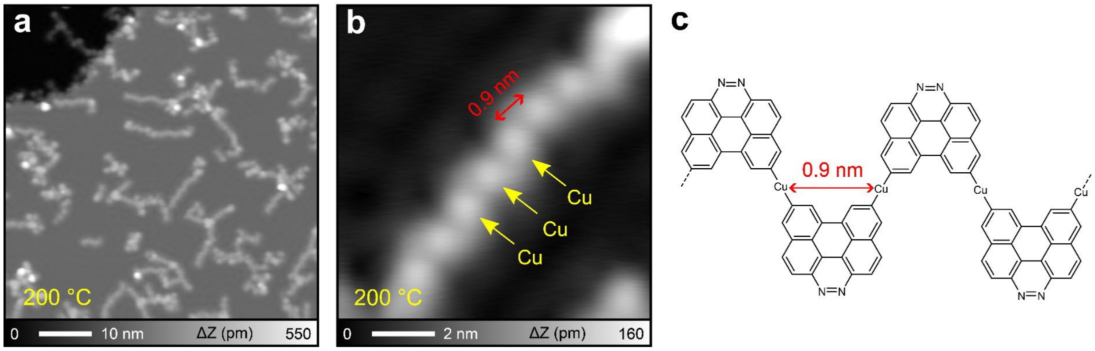

Figure S5. On-surface reaction of **1** on Cu(111). (a) Large-scale STM topography of the sample after annealing at 200 °C. (b) Close-up view of an organometallic chain and (c) the corresponding chemical structure. Measurement parameters: V = 200 mV and I = 40 pA in (a). V = 200 mV and I = 300 pA in (b).

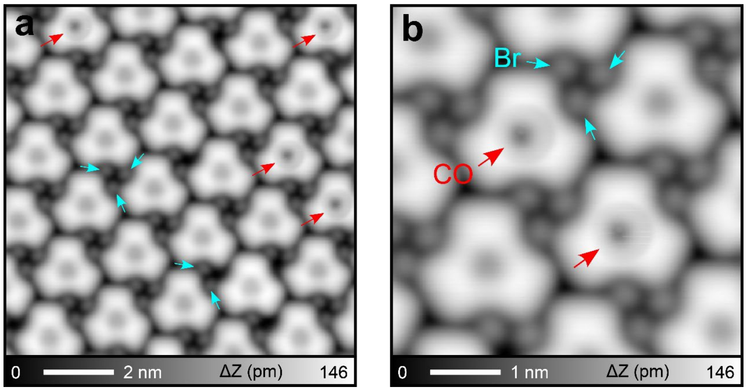

Figure S6. STM topographies of nitrogen-doped NGs **4** and Br atoms after dosing CO molecules on Ag(111): (a) Large scale and (b) Small scale. CO molecules (indicated by red arrows) can adsorb at the center of NGs **4**. Measurement parameters: V = 200 mV and I = 10 pA in (a). V = -200 mV and I = 10 pA in (b).

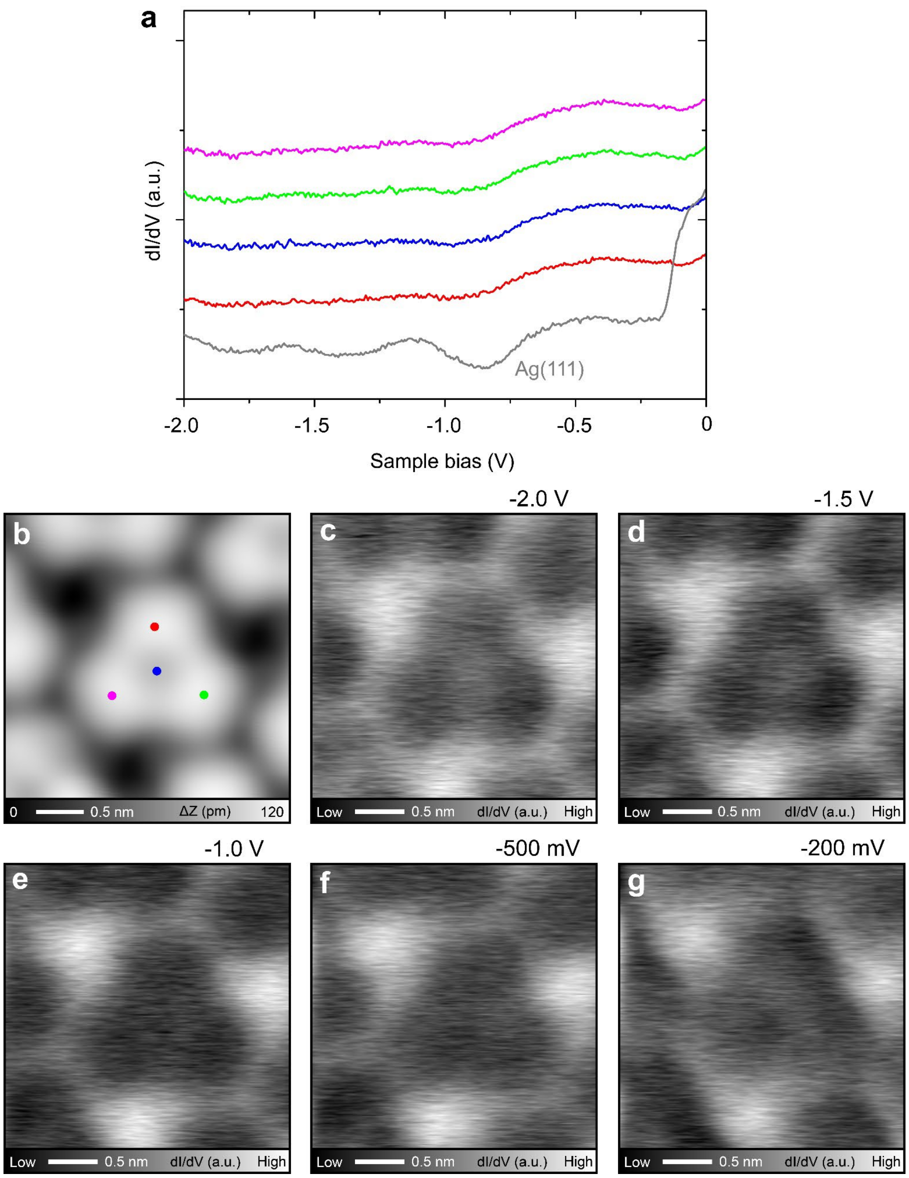

Figure S7. STS measurement of the nitrogen-doped NG **4** in negative bias voltage range. (a) d*I*/d*V* curves recorded above individual **4** (color dots indicated in (b)) and bare Ag(111) surface (gray line). (b) STM topography of the measured **4** on Ag(111). (c-g) Constant current d*I*/d*V* maps taken at bias voltages of (c) -2.0 V, (d) -1.5 V, (e) -1.0 V, (f) -500 mV and (g) -200 mV. Measurement parameters: V = 200 mV and I = 60 pA in (b). V = -2.0 V, I = 150 pA, V_{ac} = 10 mV for STS in (c). V = -1.5 V, I = 130 pA, V_{ac} = 10 mV in (d). V = -1.0 V, I = 110 pA, V_{ac} = 10 mV in (e). V = -500 mV, I = 100 pA, V_{ac} = 10 mV in (f). V = -200 mV, I = 60 pA, V_{ac} = 10 mV in (g).

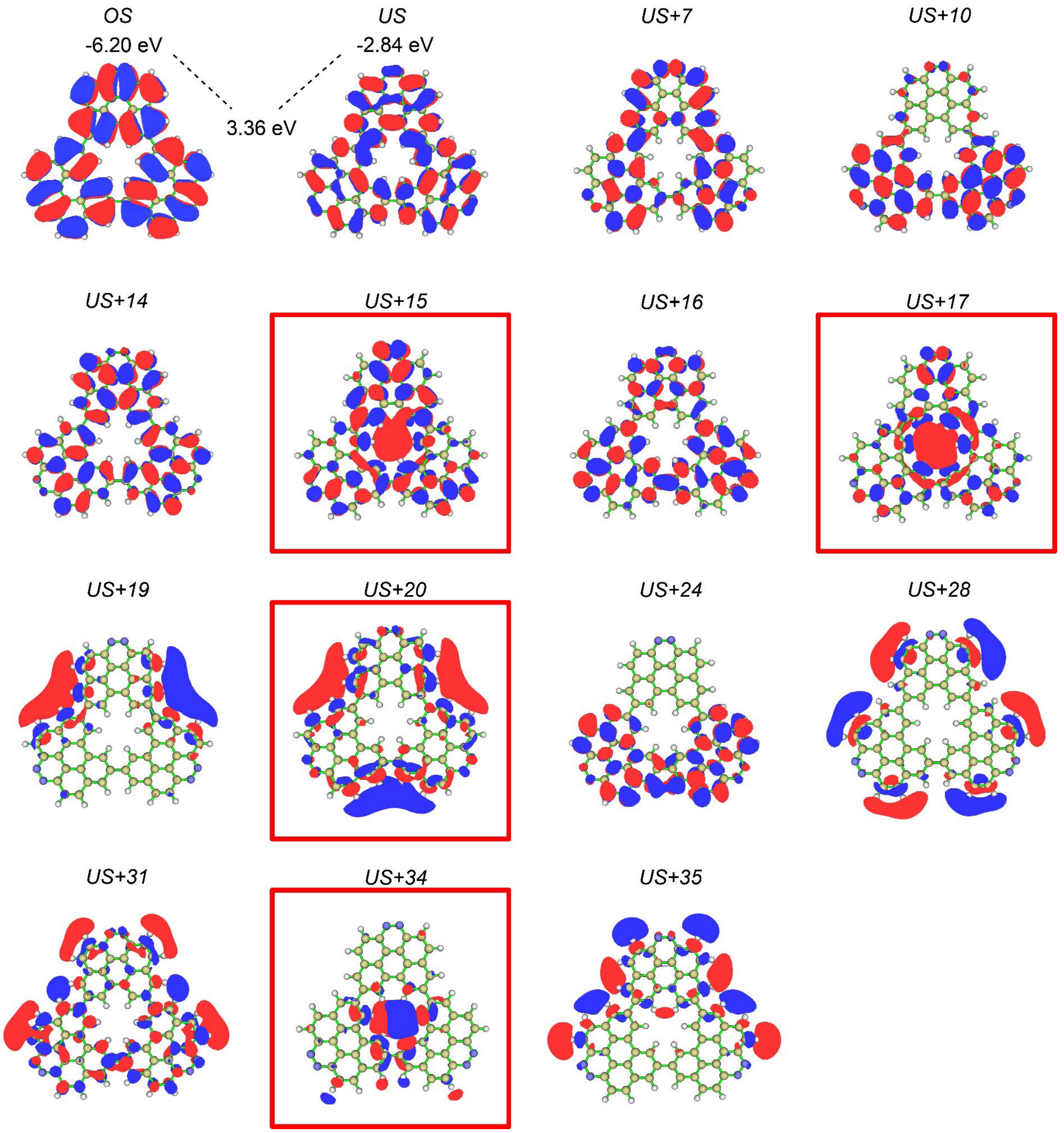

Figure S8. DFT calculations for the occupied and unoccupied molecular states of free-standing **4**. These maps show the unoccupied molecular and hybrid states. The calculated band gap of free-standing **4** is 3.36 eV.

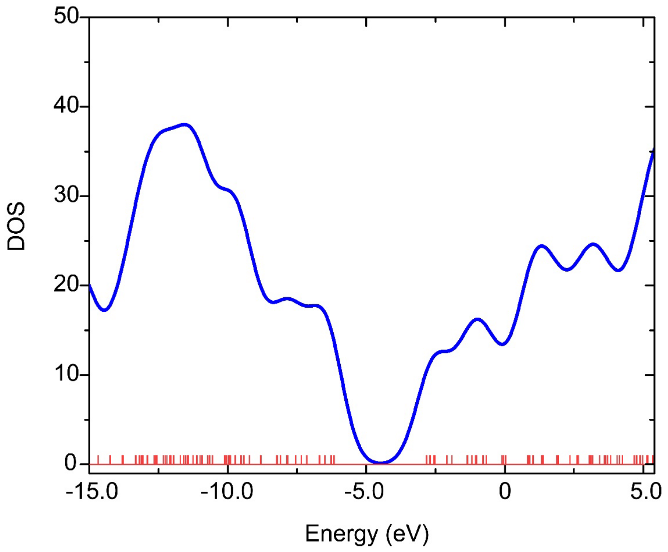

Figure S9. Calculated total density of states (DOS) for free-standing **4**. The small red vertical lines represent energy levels of molecular states. Blue curve indicates the DOS for total atoms of **4**.

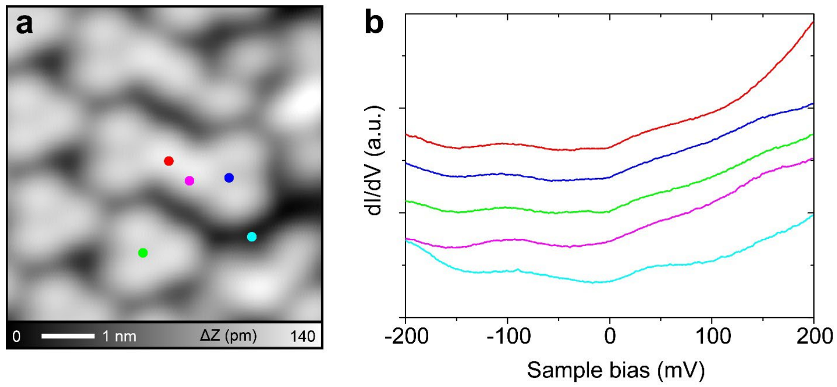

Figure S10. STS measurement of the nitrogen-doped NG dimer around Fermi level. (a) STM topography of the dimer and the monomer. (b) d*I*/d*V* curves taken above the junction of the N-NG dimer and other sites as references. Measurement parameters: $V = 100$ mV and $I = 10$ pA in (a). $V = 200$ mV, $I = 300$ pA, $V_{ac} = 10$ mV for STS in (b).

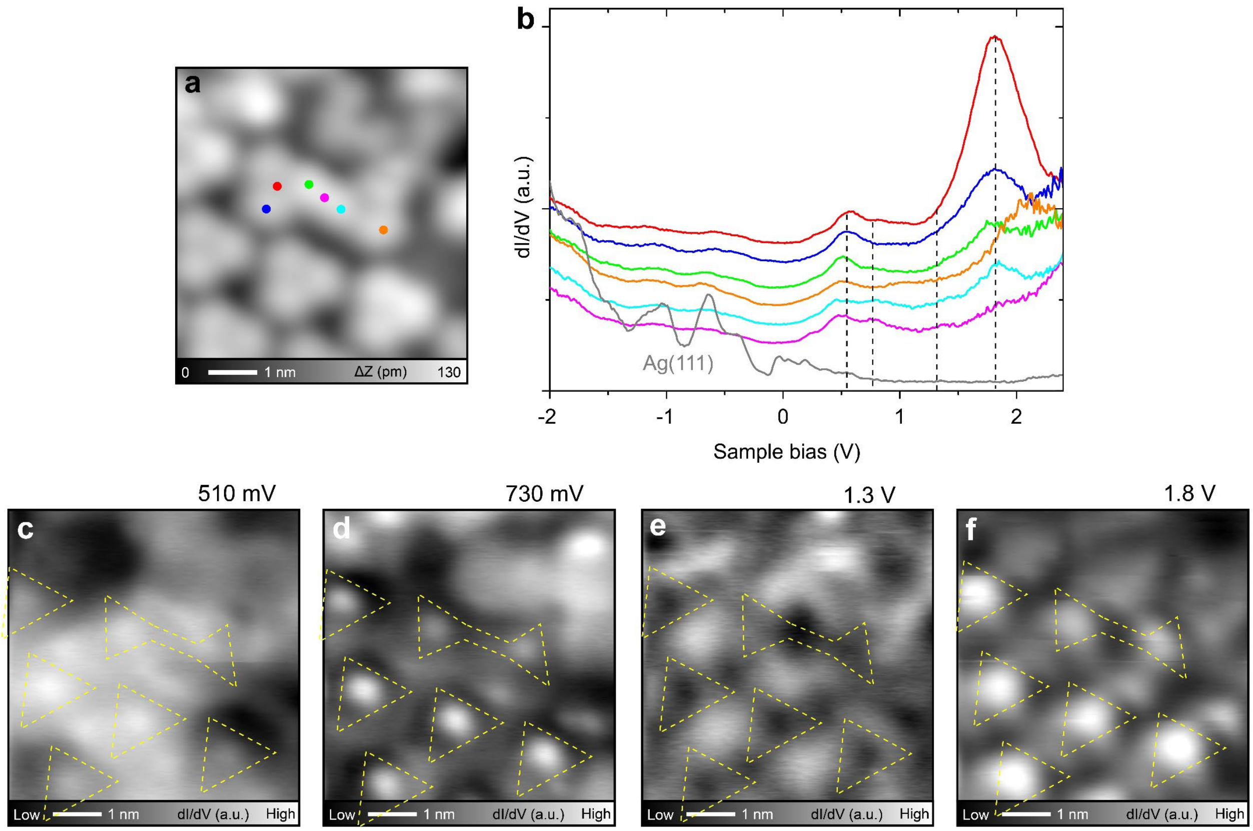

Figure S11. STS measurement of the nitrogen-doped NG dimer. (a) STM topography of the dimer and the monomer. (b) dI/dV curves recorded above the dimer (color dots indicated in (a)) and bare Ag(111) surface (gray line). (c-f) Constant current dI/dV maps taken at bias voltages of (c) 510 mV, (d) 730 mV, (e) 1.3 V and (f) 1.8 V. Measurement parameters: V = 200 mV and I = 10 pA in (a). V = 1 V, I = 120 pA, V_{ac} = 10 mV for STS in (b). V = 510 mV, I = 150 pA, V_{ac} = 10 mV in (c). V = 730 mV, I = 150 pA, V_{ac} = 10 mV in (d). V = 1.3 V, I = 150 pA, V_{ac} = 10 mV in (e). V = 1.8 V, I = 150 pA, V_{ac} = 10 mV in (f).

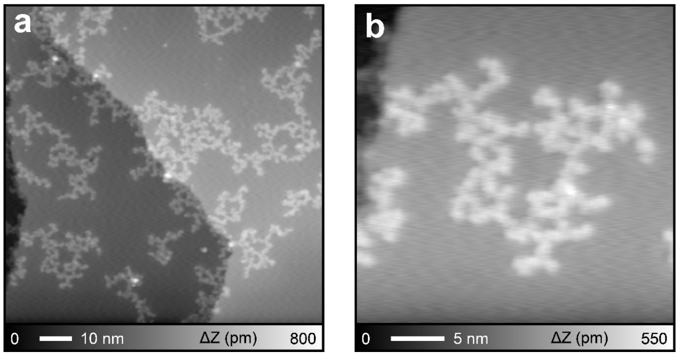

Figure S12. STS measurement of the Ag(111) surface after annealing at 350 °C for 5 min. (a) Large scale STM topography. (b) Small scale STM topography. Measurement parameters: $V = 200$ mV and $I = 6$ pA in (a). $V = 200$ mV and $I = 10$ pA in (b).
