# Supplementary material for: On-surface synthesis of nitrogen-doped nanographene with an [18]annulene pore on Ag(111)
Source: Commun Chem. 2023 Oct 20;6:228. doi: 10.1038/s42004-023-01023-z (PMC10589310; doi:10.1038/s42004-023-01023-z)
Supplement: Supplementary file 2 — Description of Additional Supplementary File [file 42004_2023_1023_MOESM2_ESM.pdf]

# Description of Additional Supplementary

**File name:** Supplementary data 1

**Description:** XYZ coordinates for the calculated species
